# Distinct Contributions of Astrocytes and Pericytes to Neuroinflammation Identified in a 3D Human Blood-Brain Barrier on a Chip

**DOI:** 10.1371/journal.pone.0150360

**Published:** 2016-03-01

**Authors:** Anna Herland, Andries D. van der Meer, Edward A. FitzGerald, Tae-Eun Park, Jelle J. F. Sleeboom, Donald E. Ingber

**Affiliations:** 1 Wyss Institute for Biologically Inspired Engineering, Harvard University, Boston, Massachusetts, United States of America; 2 Harvard John A. Paulson School of Engineering and Applied Sciences, Cambridge, Massachusetts, United States of America; 3 Department of Pathology, Harvard Medical School, Boston, Massachusetts, United States of America; 4 Vascular Biology Program, Department of Surgery, Boston Children’s Hospital, Boston, Massachusetts, United States of America; Hungarian Academy of Sciences, HUNGARY

## Abstract

Neurovascular inflammation is a major contributor to many neurological disorders, but modeling these processes *in vitro* has proven to be difficult. Here, we microengineered a three-dimensional (3D) model of the human blood-brain barrier (BBB) within a microfluidic chip by creating a cylindrical collagen gel containing a central hollow lumen inside a microchannel, culturing primary human brain microvascular endothelial cells on the gel’s inner surface, and flowing medium through the lumen. Studies were carried out with the engineered microvessel containing endothelium in the presence or absence of either primary human brain pericytes beneath the endothelium or primary human brain astrocytes within the surrounding collagen gel to explore the ability of this simplified model to identify distinct contributions of these supporting cells to the neuroinflammatory response. This human 3D BBB-on-a-chip exhibited barrier permeability similar to that observed in other *in vitro* BBB models created with non-human cells, and when stimulated with the inflammatory trigger, tumor necrosis factor-alpha (TNF-α), different secretion profiles for granulocyte colony-stimulating factor (G-CSF) and interleukin-6 (IL-6) were observed depending on the presence of astrocytes or pericytes. Importantly, the levels of these responses detected in the 3D BBB chip were significantly greater than when the same cells were co-cultured in static Transwell plates. Thus, as G-CSF and IL-6 have been reported to play important roles in neuroprotection and neuroactivation *in vivo*, this 3D BBB chip potentially offers a new method to study human neurovascular function and inflammation *in vitro*, and to identify physiological contributions of individual cell types.

## Introduction

The blood vessels in the brain are of major physiological importance because they maintain the blood-brain barrier (BBB), support molecular transport across this tight barrier, control local changes in oxygen and nutrients, and regulate the local immune response in the brain [1]. Neurovascular dysfunction also has been linked to a wide spectrum of neurological disorders including multiple sclerosis [2], Alzheimer’s disease [3], and brain tumors [4]. Due to its relevance for neurophysiology and pathophysiology, more realistic models of the human neurovascular niche are needed to advance fundamental and translational research, as well development of new and more effective therapeutics.

The BBB is formed by the continuous brain microvascular endothelium, its underlying basement membrane, pericytes that tightly encircle the endothelium, and astrocytes in the surrounding tissue space that extend their cell processes towards the endothelium and insert on the basement membrane [5]. Together, these cells maintain a highly selective permeability barrier between the blood and the brain compartments that is critical for normal brain physiology. Importantly, the pericytes and astrocytes convey cues that are required for normal function and differentiation of the brain microvascular endothelium [6, 7], and all three cell types—endothelial cells, pericytes, and astrocytes—are required for maintenance of the normal physiology of the neurovasculature and maintenance of BBB integrity *in vivo* as well as *in vitro* [5, 8]. Astrocytes also have been shown to display a large number of receptors involved in innate immunity, and when activated, to secrete soluble factors mediating both innate and adaptive immune responses [9]. Brain pericytes have likewise been demonstrated to respond to inflammatory stimuli resulting in release of pro-inflammatory cytokines [10, 11]. However, the complex interaction between these cell types and the microvascular endothelium make it extremely difficult to analyze their individual contribution to neuroinflammation *in vivo*.

Thus, in the present study, we set out to develop an *in vitro* model of the human BBB that would permit analysis of the independent contributions of human brain microvascular endothelium, pericytes and astrocytes to the response of the BBB to inflammation stimuli. The inflammatory effects of various stimuli, including TNF-α [12], lipopolysaccharide (LPS) endotoxin [13], nanoparticles [14], and HIV-virions [15] have been studied previously using static BBB models with non-human [12–14] and human [15] cells cultured in Transwell plates. Studies with these models have also demonstrated that both astrocytes and pericytes can influence the barrier function of the BBB under static conditions [16, 17]. But given inevitable species differences between humans and animal models in terms of species-specific efflux transporter activity, tight junction functionality and cell-cell signaling [18, 19], it is critical to carry out studies using normal human brain microvascular cells to recapitulate human brain microvascular physiology. In fact, interactions between human primary astrocyte and human brain microvascular cells have been analyzed in static Transwell cultures, and the results of these studies have shown correlations with *in vivo* studies for radiotracer permeability profiles and barrier function [20, 21]. However, hemodynamic forces and the physical tissue microenvironment are also known to contribute significantly to microvascular function [22, 23]. Thus, to best model the BBB *in vitro*, it is important to mimic these key physical features of the brain capillary microenvironment, including fluid flow, extracellular matrix (ECM) mechanics, and the cylindrical geometry of normal brain microvessels. BBB cell culture models based on semi-permeable, synthetic hollow-fibers with a blood vessel-like geometry and fluid flow have been developed [24, 25], and more recently, microfluidic models of the BBB have been reported that enable co-culture of endothelium with pericytes, astrocytes or neurons while being exposed to fluid flow and low levels shear stress [26–34]. However, all of these *in vitro* BBB models utilized rigid ECM substrates that have stiffness values orders of magnitude higher than those observed in living brain microvessles (i.e., ~1 GPa for ECM-coated cell culture plastic versus ~1 kPa *in vivo*) [35] and none cultured neurovascular cells in a normal cylindrical vascular conformation. Microfluidic models have been developed that contain more flexible ECM gels and reconstitute 3D hollow vessel-like structures [36–40], but the only reported studies that use such techniques to model the BBB used non-human endothelium [41, 42]. Human brain endothelial cells, pericytes and astrocytes also have been maintained in close juxtaposition in spheroid cultures, but vessels do not form in these structures, and instead they resemble endothelium-lined spheres [43]. In the present study, we therefore set out to develop a 3D microfluidic model of a hollow human brain microvessel that contains closely apposed primary microvascular endothelial cells, pericytes and astrocytes isolated from human brain, specifically to analyze the contribution of the individual cell types to neurovascular responses to inflammatory stimuli. We demonstrate the utility of this new organ-on-a-chip model for studying neurovascular inflammation by measuring cytokine release induced by adding tumor necrosis factor-alpha (TNF-α) as an inflammatory stimulus, and analyzing how the presence of astrocytes and pericytes independently contribute to this response. As this 3D BBB-on-a-chip permits analysis of the contributions of individual cell types to neuropathophysiology, it may be useful for studies focused on the mechanisms that underlie inflammation in the human brain as well as related screening of neuroactive therapeutics.

## Materials and Methods

### Cell culture

Human brain microvascular endothelial cells (hBMVECs) and human brain pericytes, both derived from cortex, were obtained from Cell Systems (Kirkland, WA USA) and maintained with CSC complete medium (Cell Systems, Kirkland, WA USA) on regular tissue culture flasks coated with Attachment factor (Cell Systems, Kirkland, WA USA). Human astrocytes of cortical origin were obtained from ScienCell (San Diego, CA USA) and maintained in Astrocyte medium (ScienCell, San Diego, CA USA). All cells were used at passage 3 to 8.

### Microfluidic chips, fabrication and pre-treatment

Molds for microfluidic channels with a width, height and length of 1 mm, 1 mm and 20 mm, respectively, were designed with SolidWorks software (Dassault Systèmes SOLIDWORKS Corp.) and produced by Fineline stereolithography (Proto Labs, Inc.). Microfluidic devices were subsequently produced by soft lithography. Briefly, a degassed 10:1 base:crosslinking mix of Sylgard 184 polydimethylsiloxane (PDMS, Dow Corning, Inc.) was poured onto the mold and allowed to crosslink at 80°C for 18 hours. Inlets and outlets of 1.5 mm diameter were punched in the molded PDMS and the device was bonded to a 100 μm layer of spincoated PDMS by pre-treating with oxygen plasma at 50 W for 20 seconds in a PFE-100 (Plasma Etch, Inc.) and then pressing the surfaces together. After baking at 80°C for 18 hours, devices were again treated with oxygen plasma (30 seconds, 50 W) and silanized by immediately filling them with 10% (v/v) of (3-aminopropyl)-trimethoxysilane (Sigma) in 100% ethanol and incubating at room temperature for 15 minutes. Devices were then flushed with 100% ethanol, followed by water and ethanol and subsequently dried at 80°C for 2 hours. Subsequently, the surfaces were further functionalized by filling the devices with 2.5% glutaraldehyde (Electron Microscopy Services, Inc.). After incubating for 15 minutes, the devices were rinsed extensively with deionized water and ethanol and were baked for 2 hours at 80°C. The Schiff bases formed on proteins after glutaraldehyde immobilization are stable without further reduction, as has been demonstrated in surface-protein conjugation [44].

### Viscous fingering to generate lumens in collagen gels

The viscous fingering procedure was performed as previously reported [39], with slight modifications. Using the published method, we found that the collagen gel tended to delaminate from the PDMS microchannel surface, and so we functionalized the PDMS surface in a three-step process involving oxygen plasma treatment, amino-silane conjugation and glutaraldehyde derivatization. This treatment improved the stability of the PDMS-collagen interaction such that no delamination was observed, and this protocol allowed the chips to remain stable for more than 7 days with no apparent degradation.

All devices pre-treated in this manner were kept on ice and filled with ice-cold 5 mg/ml rat tail collagen I (Corning), mixed and neutralized as per the manufacturer’s instructions. Immediately after filling the device with the collagen solution, a 200 μl pipette tip with 100 μl of ice-cold culture medium was inserted in the inlet. The medium was allowed to flow through the viscous collagen solution by hydrostatically driven flow and the devices were subsequently incubated at 37°C to allow the formation of collagen gels. Alternatively, to correlate hydrostatic pressure with lumen diameter, the devices were connected to a liquid reservoir that could be placed at different heights. The pressure values presented here were calculated as the difference in height between the meniscus of the liquid in the reservoir and the inlet of the chip. After collagen gelation by incubating for 30 minutes at 37°C, the devices were rinsed extensively with pre-warmed culture medium and stored in a cell culture incubator for 18 hours. In our studies, we used an input pressure of 2.6 cm H_2_O (0.26 kPa) to form the lumen, and a minimal pressure of 1.5 cm H_2_O (0.15 kPa) was needed to initiate formation of the finger in a collagen gel in the 1×1 mm channel. Microchannels with smaller dimensions, down to 300 × 300 μm were evaluated, but these yielded significantly lower success rates due to increased clogging of lumens with collagen or complete removal of the gels due to the need to apply increased pressures.

### Cell culture in three-dimensional gels

Human astrocytes were incorporated in the bulk of the collagen by mixing in a final concentration of 3×10^6^ cells/ml in the gel. Following 18 hours of incubation of devices in a cell culture incubator, sequential seeding of pericytes and hBMVECs was carried out to line the cylindrical lumen with these two cells types. Pericytes were seeded into the devices at 0.8×10^6^ cells/ml in two rounds, where the devices were put upside down in the first seeding round. An incubation period of 30 min was allowed between the seeding steps. 30 minutes after pericyte seeding hBMVECs were seeded at 2.4×10^6^ cells/ml under flow for 20 seconds (120 μl/min; ~1 dyne/cm^2^ shear stress) using the described two-step seeding method to obtain a lumen lined with an endothelial monolayer. One hour after final cell seeding, medium was exchanged by hydrostatically driven flow. The chips were maintained under static conditions in a cell culture incubator with the cell culture medium being exchanged over a period of 5 minutes every 24 hours using hydrostatically-driven flow at 120 μl/min (~1 dyne/cm^2^ shear stress). Once a confluent monolayer formed, which was typically after 72 hours, 250 μM of a cell-permeable cyclic adenosine monophosphate, 8-CPT-cAMP, (Abcam) and 17.4 μM of the phosphodiesterase inhibitor Ro 20–1724 (Santa Cruz Biotech) was added to the medium, which was exchanged periodically as described above. We did not culture the cells under continuous flow for the 5 days of culture (although we did this for analytical studies) because to get a realistic shear stress in the range of 1–10 dyne/cm^2^, we would need flow rates in the range of 600 ml/hour, which would be exorbitantly expensive.

### Permeability assay

We cannot measure TEER to evaluate the barrier function of our 3D BBB Chip due to the difficulty of placing electrodes on opposite sides of the endothelium with a surrounding solid ECM gel and ensuring an even electrical field given the device geometry. So instead we evaluated the permeability coefficient for small molecular (3 kDa) fluorescent dextran. Devices were cultured for 120 hours before they were mounted on a Zeiss Axio Observer microscope, with a 5× air objective, numerical aperture 0.14 with an Evolve EMCCD camera. Culture medium with 5 μg/ml dextran 3 kDa-Alexa488 (Life Technologies) was continuously infused in the microfluidic chips at 5 ml/hour with a syringe pump (~0.7 dyne/cm^2^ shear stress) and fluorescent images were recorded every 3 seconds over 2 hours. Apparent permeability (P_app_) was calculated by analyzing total fluorescence intensity in an area of 1 mm by 1 mm and then applying P_app_ = (1/ΔI) (dI/dt)_0_ (r/2), where ΔI is the increase in total fluorescence intensity upon adding labeled dextran, (dI/dt)_0_ is the initial rate of increase in intensity as dextran diffuses out of the tube into the surrounding gel, and r is the radius of the tube [45]. (dI/dt)_0_ was determined by analyzing the linear increase in fluorescence signal during 5 minutes. Control measurements for the recorded intensity demonstrated a linear response of the detector in the range of 5 μg/ml dextran 3 kDa-Alexa488. The wide depth of field of the objective allowed for collection of all fluorescent signal from the 1 mm high channel. Control measurements confirmed that the fluorescence signal from microchannels of heights 200 μm-1000 μm filled with 5 μg/ml dextran 3 kDa-Alexa488 increased linearly with channel height. The permeability measurement method cannot be applied to the bare collagen lumens or to cultures of astrocytes or pericytes alone because the diffusion of the 3 kDa dextran is too fast to reliably establish the intensity step ΔI.

### Transwell cell culture

24-well Transwell inserts (Corning), 0.4 μm, polyethylene terephthalate membrane, were coated with rat-tail collagen I (Corning) at 100 μg/ml in phosphate-buffered saline for 2 hours. The inserts were inverted and pericytes or astrocytes were seeded at 6.25×10^3^ cells per insert. After 2 hours of incubation, the inserts were placed in 24-well plates and seeded with hBMVEC at 2.5×10^4^ cells per insert. Transendothelial electrical resistance (TEER) values were measured after 120 hrs of culture using an EndOhm (WPI) and chopstick electrodes. Paracellular diffusion was assayed 5 minutes after adding dextran 3 kDa-Alexa488 (100 μg/ml) to the apical chamber and using a Synergy Neo platereader (BioTek).

### Inflammatory stimulation and analysis of cytokine release

Microfluidic chips and Transwell inserts were cultured for 72 hours, followed by incubation in CSC complete medium with fetal bovine serum reduced from 10% to 2% for 18 hours. Microfluidics chips were stimulated with TNF-α (Sigma Aldrich) at 50 ng/ml in CSC complete medium with 2% serum for 6 hrs (5 min flow at 120 μl/min corresponding to ~1 dyne/cm^2^, followed by static conditions). Transwells were stimulated on the apical and the basal side. Following thorough rinsing of microfluidic chips under continuous flow (120 μl/min; ~1 dyne/cm^2^) and batch washes of Transwell plates with CSC complete medium with 2% serum, conditioned medium from the chips was collected continuously for 1 hour at 100 μl/hr (~0.01 dyne/cm^2^) using syringe-driven flow; medium from the apical compartment was collected from Transwells after 1 hour. The cytokine release profile was assayed with the Bio-Plex Pro Human Cytokine 17-plex Assay (BioRad) in a Bioplex 3D system (BioRad), and the resulting cytokine release profiles were normalized to cell culture area in 3D BBB chips versus Transwells.

### Fixation, staining and imaging

Microfluidic chips were cultured for 96 hours followed by rinsing in phosphate-buffered saline and fixation in 4% paraformaldehyde (Sigma) for 20 minutes at room temperature. Cell-free devices were fixed 30 minutes after collagen gelation. Immunocytochemistry was carried out after permeabilization in phosphate-buffered saline with 0.1% Triton X-100 (Sigma) and blocking for 30 minutes in 10% goat serum in phosphate-buffered saline with 0.1% Triton-X 100. The following primary antibodies were used for immunocytochemistry experiments: rabbit anti-glial fibrillary acidic protein (GFAP) (Millipore, 1:100), mouse anti-vascular endothelial (VE)-cadherin (Abcam, 1:100), mouse anti-PECAM (eBiosciences, 1:100), mouse anti-zona occludens-1 (ZO-1) (Invitrogen 1:100), rabbit anti-alpha-smooth muscle actin (SMA) (Sigma, 1:100) and mouse anti-collagen IV (Millipore). The secondary antibodies were anti-rabbit or anti-mouse IgG conjugated with Alexa Fluor-488, Alexa Fluor-555, or Alexa Fluor-647 (Invitrogen). Hoechst (10 mg/ml, Invitrogen) was used at a dilution of 1:5000 for nuclei staining. For staining of F-actin, Alexa Fluor-488-phalloidin or Alexa Fluor-647-phalloidin (Invitrogen) were used at dilution of 1:30. Imaging was carried out using a Leica SP5 X MP Inverted Laser Scanning Confocal Microscope with a 25× water immersion objective and a Zeiss Axio Observer microscope. Conventional confocal imaging was carried out with a 405 laser diode, an Argon laser and a tunable white laser. Second harmonic generation was carried out using two-photon excitation at 810 nm and detecting emitted light through a 400–410 nm bandpass filter. Image processing was done using Huygens deconvolution and stitching for tiled images (SVI), Imaris (Bitplane) and ImageJ. The low objective flatness gives a Gaussian intensity profile over each recorded image, which becomes apparent in stitched images 2c and 2k.

### Statistics

All experiments were carried out at n = 3–7; exact numbers are mentioned per experiment in figure captions. Prism (GraphPad) was used for one-way ANOVA analysis with Bonferroni post-test. **** denotes p< 0.0001, *** denotes 0.0001<p <0.001, ** denotes 0.001<p <0.01, * denotes 0.01<p <0.05. For significance testing between two conditions a non-paired student’s t-test was used.

## Results and Discussion

### Engineering of the 3D BBB chip

**Fig 1:**
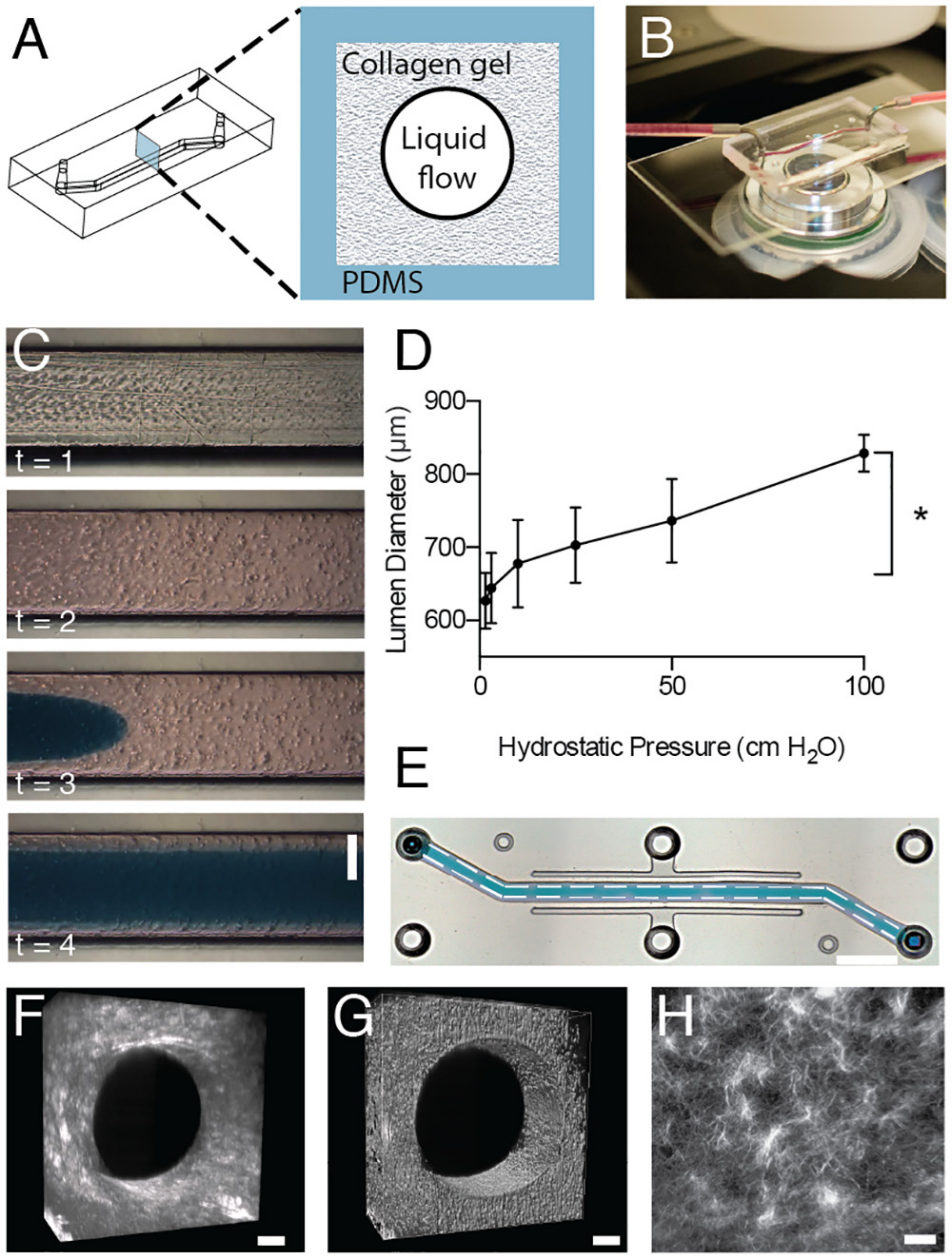
The pressure-driven viscous fingering method used to generate the cylindrical collagen gel in the 3D BBB chip. A) Schematic diagram of the PDMS structure used to generate the 3D BBB chip (left) and an illustration of a cross-section through the chip showing the PDMS channel containing the collagen gel made with viscous fingering and a central lumen (right). B) Photograph of the 3D BBB chip on the stage of an inverted microscope. C) Time-lapse images of the fingering method showing the microchannel before (t = 1) and after infusion of a neutralized collagen gel containing dispersed human astrocytes (t = 2), which was then followed by injection of a low viscosity liquid (dyed blue here) driven by hydrostatic pressure to initiate “finger” formation in the center of the gel (t = 3), and eventually a continuous hollow cylindrical lumen throughout the length of the device (t = 4). The time course from t = 1 to 4 is user dependent but normally less than 30 sec (bar, 500 μm). D) Graph showing the correlation between the hydrostatic pressures used to drive the fingering process and the resulting lumen diameter (* p<0.05 Student’s t-test, n = 3). E) Low magnification micrograph of an entire device containing a lumen filled with blue fluid, formed as described in C (dashed lines, delineate the edges of the channel; black dotted rectangle indicates where images shown in F and G were recorded (bar, 3 mm). F) Second harmonic generation image of the collagen distribution in the 3D BBB chip, and an intensity generated voxel illustration of the lumen based on this information (G) (bar, 100 μm). H) High magnification of the second harmonic generation image showing of collagen microstructure in the cylindrical gel within the 3D BBB chip (bar, 50 μm).

To build a 3D BBB chip containing a hollow endothelium lined microvessel surrounded by a compliant ECM, we first formed a cylindrical collagen gel within a single square-shaped microchannel (1 mm high × 1 mm wide × 2 cm long) (Fig 1A) in an optically clear polydimethysiloxane (PDMS) chip mounted on a standard glass microscope slide (Fig 1B) using soft lithography, as previously described [39]. The cylindrical collagen gel was formed using a published viscous fingering method [46] by first filling the channel with a solution of type I collagen (5 mg/ml), applying hydrostatically-controlled medium flow (by varying the height of the fluid reservoir) to finger through this viscous solution, and finally incubating the chips at 37°C to promote gelation (Fig 1C). The entire process takes about 30 seconds and results in the creation of a well-defined lumen with a diameter of ~600 to 800 μm protruding all the way through the 2 cm long channel of the microfluidic chip (Fig 1E). The dimensions of the lumen are controlled by the channel dimensions and by the differences in viscosity and density between the displacing and displaced liquid [39, 47, 48]. Theoretically, an increased pressure will produce a higher tip velocity of the finger, which should lead to a narrower finger (smaller lumen diameter) [47]; however, we empirically found that progressively increasing the hydrostatic pressure of the injected medium resulted in a concomitant increase in lumen diameter (Fig 1D). It is possible that the positive correlation between input pressure and lumen diameter we observed here might be due to increased shearing of collagen at high flow velocities in the channel directly after the lumen has formed.

Use of second harmonic generation imaging revealed that the cylindrical collagen gel formed in the microchannel with this viscous fingering method contained a homogenous, loose, fibrillar collagen matrix with a low number of points of high fibril density located preferentially along the wall of the PDMS channel (Fig 1F–1H). This loose, homogenous ECM network is more similar to that observed in the subendothelial space in the brain than the planar ECM-coated substrates used in past BBB chip models [26–29]. In addition, when supporting cells, such as human brain astrocytes, are suspended into the collagen solution, they evenly distribute throughout the gel as it undergoes viscous fingering and gelation in the microchannel (Fig 1C). Thus, this cylindrical collagen gel is well suited to recapitulate the supporting ECM framework of the BBB on-chip; moreover, the viscous fingering or other lumen formation methods in hydrogels [49] could be used to further explore the contributions of ECM composition and mechanics [50] in future studies.

### Structural reconstitution of the human Blood-Brain Barrier

**Fig 2:**
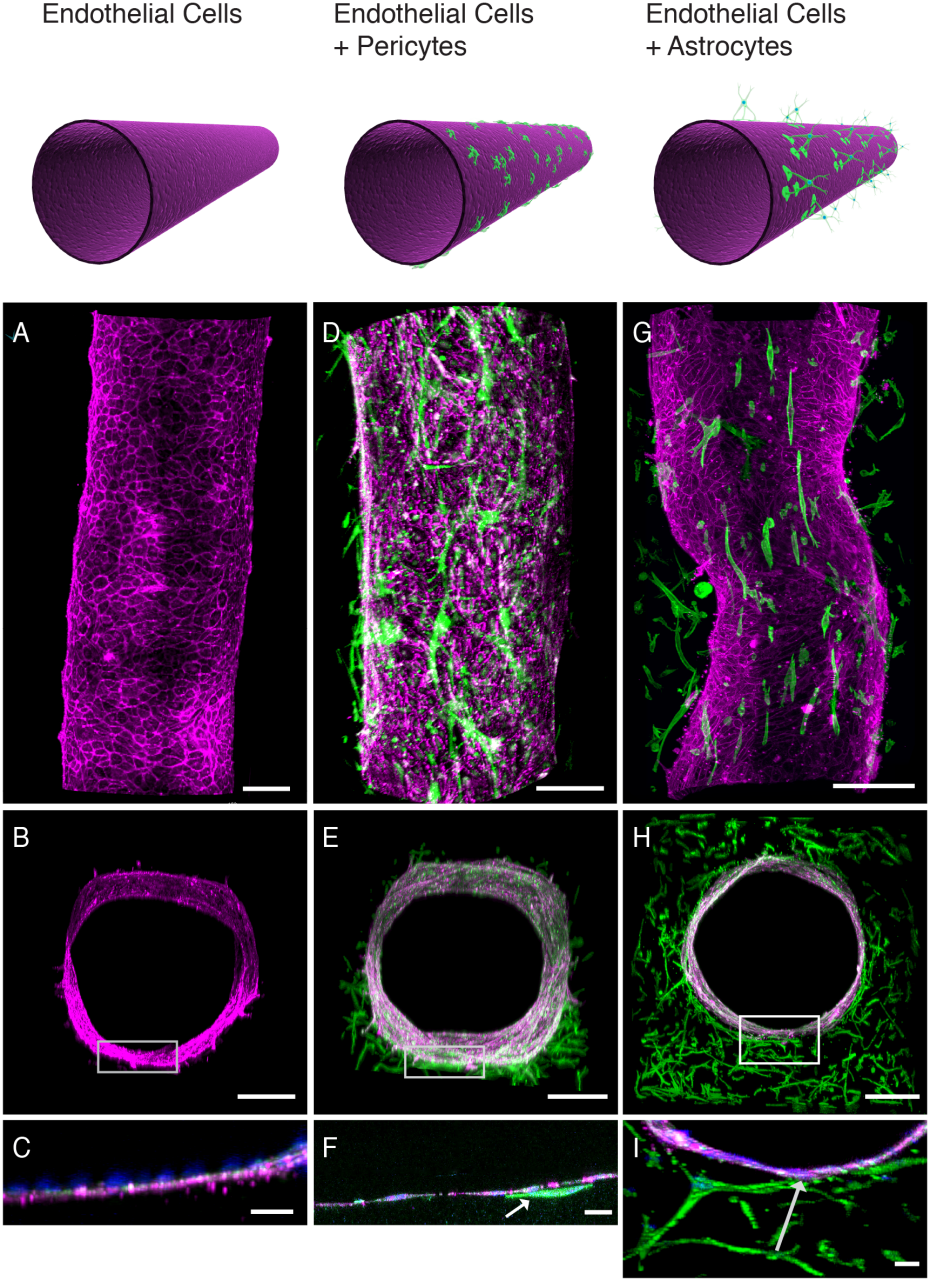
Co-culture of human brain microvascular endothelial cells, pericytes and astrocytes in the 3D BBB chip. Schematic illustrations of the cells populating the 3D vessel structures for the three experimental set-ups are shown at the top, and fluorescence confocal micrographs of the engineered brain microvessel viewed from the top (A, D, G) or shown in cross-section at either low (B, E, H) or high (C, F, I) magnification (rectangles in lower magnifications images indicate respective areas shown at higher magnification below). The fluorescence micrographs show the cell distributions in 3D BBB chips containing brain microvascular endothelium alone (A-C), endothelium with prior plating of brain pericytes on the surface of the gel in the central lumen (D-F) or endothelium with brain astrocytes embedded in the surrounding gel (G-I). High-magnification cross-sections are projections of confocal stacks (bars, 200 μm in A,B,D,E,G,H and 30 μm in C, F, I). Green indicates F-actin staining, blue represents Hoechst-stained nuclei, and magenta corresponds to VE-Cadherin staining, except for G where morphology and intensity masks were used to discriminate astrocytes (green) from endothelial cells (magenta); original image can be seen in S2 Movie. Arrows indicate contact points between endothelium and pericytes (F) or astrocytes (I).

**Fig 3:**
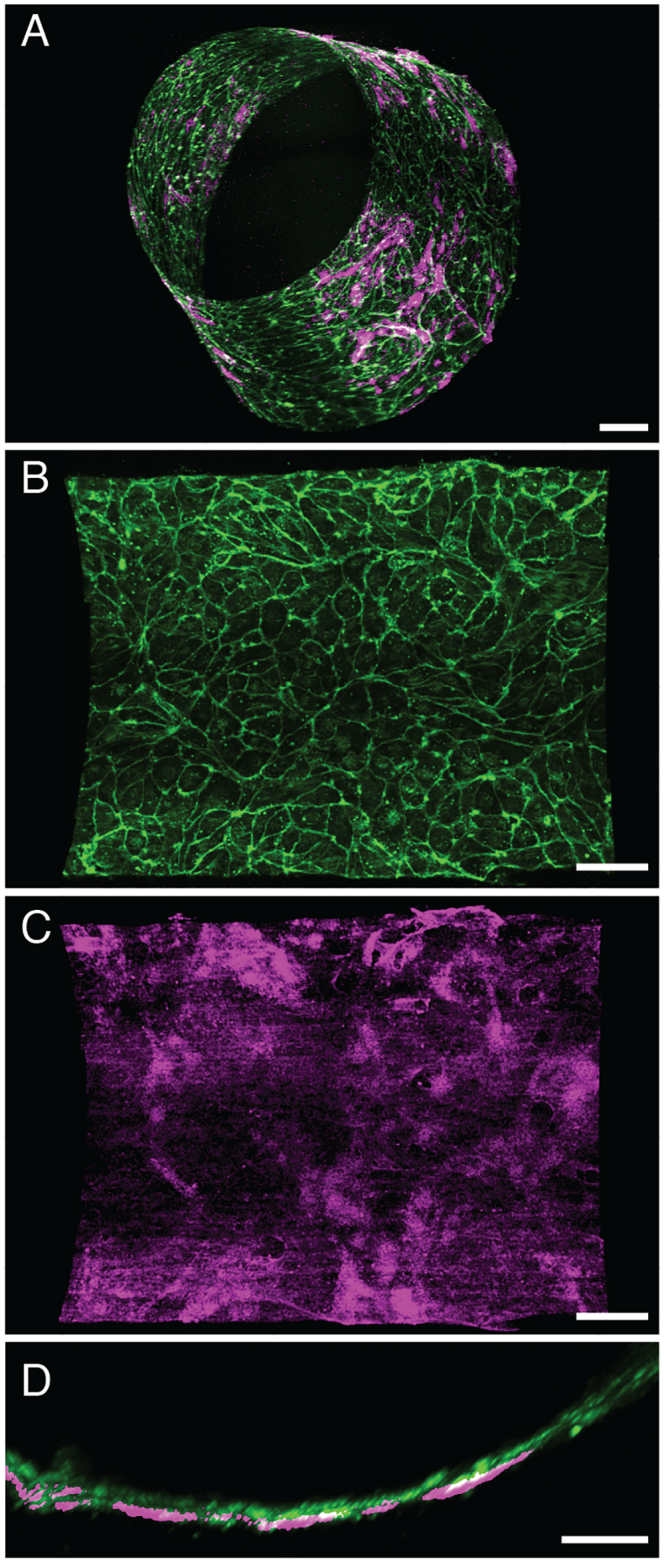
Production of an abluminal basement membrane by brain endothelial cells in the 3D BBB chip. A) Perspective view of a 3D reconstruction of a confocal fluorescence micrograph showing a monolayer of brain microvascular endothelial cells lining the lumen of a engineered vessel in the 3D BBB chip (green, F-actin staining; magenta, collagen IV staining). Higher magnification views of staining for F-actin (B) and collage IV (C), and a cross-sectional view (D) showing the accumulation of a linear pattern of basement membrane collagen IV (magenta) staining beneath the F-actin (green) containing endothelial cells (bars, 100 μm in A; 80 μm in B, C; 40 μm in D).

To mimic the human BBB *in vitro*, we seeded primary human brain-derived microvascular endothelial cells on the inner surface of the cylindrical collagen gel by flowing 40 μl of a cell suspension through the lumen, stopping flow for 1 hour to allow them to attach, and then reconstituting medium flow for 5 min at a shear stress of 1 dyne/cm^2^ once every day over the 4-5 days of culture. Confocal fluorescence microscopic analysis revealed that the endothelial cells adherent to the inner surface of the collagen gel formed a continuous monolayer with continuous VE-cadherin-containing junctions, thereby creating a cylindrical endothelium-lined microvessel on-chip (Fig 2A–2C and S1 Movie). The human brain microvascular endothelial cells also express tight junctions containing ZO-1 protein (S1 Fig). The continuous endothelium followed the contours of the lumen of the collagen gel, and the endothelial cells secreted their own underlying type IV collagen-containing basement membrane along the cell-matrix interface (Fig 3) as they do *in vivo*.

We then integrated either primary human brain pericytes or astrocytes that respectively expressed α-smooth muscle actin (SMA) or glial fibrillary acidic protein (GFAP) (S1 Fig) into these engineered microvessels. These pericytes do not express endothelial-specific markers (VE-Cadherin and PECAM), nor do they form tight cell-cell junctions that could create a tight permeability barrier of its own, as indicated by the presence of clear spaces between cells (S1 Fig). To explore the contributions of pericytes, we seeded them first onto the luminal surface of the collagen gel for 30 minutes before plating the endothelial cells, and then maintained them in culture for 4–5 days. In contrast, the astrocytes were embedded in the gel solution during the viscous fingering process to distribute them throughout the surrounding collagen matrix (Fig 1C) before the endothelial cells were plated.

The pericyte seeding method resulted in effective integration of the pericytes into the engineered microvessel such that many of them located in a circumferential abluminal distribution in tight association with the basement membrane along the basal surface of the overlying endothelium (Fig 2D–2F and S2 Fig), thus closely mimicking the position they take *in vivo* [1, 7]. When the astrocytes were embedded in the collagen gels, they filled the ECM space, extended processes towards the endothelium and contacted the basement membrane at the base of the endothelium (Fig 2G–2I and S2 Movie). These cells remained viable and sustained these relationships for the entire 4–5 day course of the study.

### Cell contributions to the permeability of the engineered 3D blood-brain barrier

**Fig 4:**
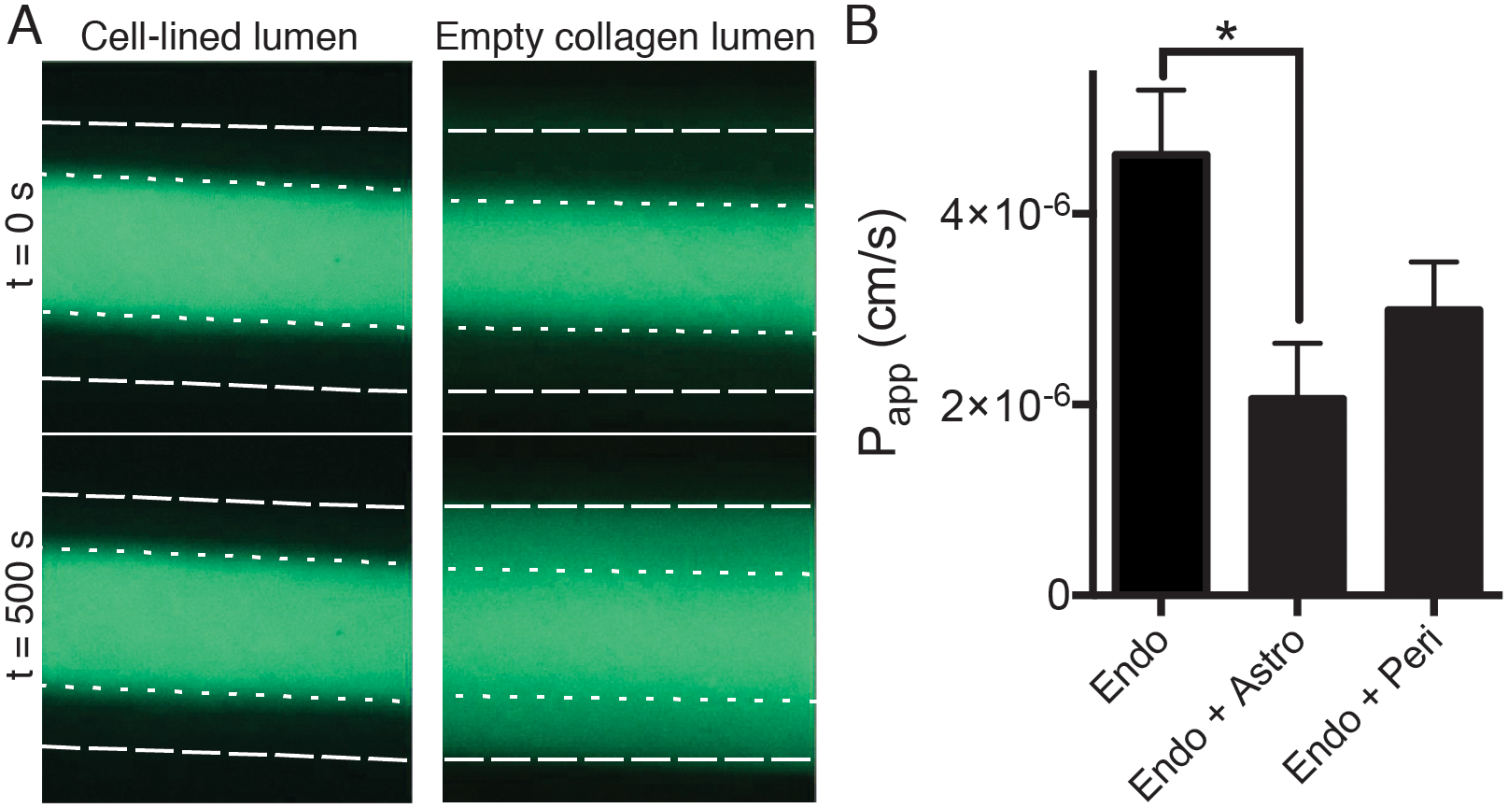
Establishment of a low permeability barrier by the engineered brain microvascular endothelium in the 3D BBB chip. A) Fluorescence micrograph of a chip containing a cylindrical collagen gel viewed from above with (left) or without (right) a lining endothelial monolayer after five days of culture (left; Cell-lined lumen) compared to a chip with an empty collagen lumen (right). The images were recorded at 0 (top) and 500 (bottom) sec after injection of fluorescently-labeled 3 kDa dextran to analyze the dynamics of dextran diffusion and visualize endothelial barrier function in the 3D BBB chip. Note that the presence of the endothelium significantly restricts dye diffusion compared to gels without cells (left versus right). B) Apparent permeabilities of the endothelium cultured in the 3D BBB chip calculated from the diffusion of 3 kDa dextran with an endothelial monolayer (Endo; n = 6), an endothelial monolayer surrounded by astrocytes (Endo+Astro; n = 3) and an endothelial monolayer surrounded by pericytes (Endo+Peri; n = 3). Error bars indicate S.E.M.; * p<0.05, Student’s t-test.

When we evaluated the paracellular permeability of the engineered microvessel lined only by human brain microvascular endothelium by continuously flowing fluorescently-labeled, low molecular weight (3 kDa) dextran through the lumen and analyzing its distribution using time-lapse microscopic imaging, we found that the presence of the human brain endothelium significantly restricted transfer of the fluorescent probe compared to control microchannels that contained the cylindrical collagen gel without any cells (Fig 4A). In control channels without cells, and in channels that contained pericytes or astrocytes but no endothelium, the fluorescent dextran quickly diffused through the collagen gel and reached the walls of the channel within 500 seconds, whereas it remained completed restricted to the lumen of the endothelium-lined vessel at this time, which exhibited an apparent permeability of 4 ×10^−6^ cm/s (Fig 4A). Importantly, the permeability of the endothelium-lined vessel was reduced even further when either astrocytes or pericytes were co-cultured with the endothelium, with co-cultures synergistically improving barrier function, producing apparent permeabilities in the range of 2 to 3 ×10^−6^ cm/s (Fig 4B), which are similar to values previously measured in other *in vitro* BBB models that have been created with rat, mouse, bovine or immortalized human cells [27, 51–54]. In contrast, when permeability of monocultures and co-cultures of the same cells cultured in Transwell plates were measured using 3 kDa dextran, values were significantly higher (from 1×10^−5^ to 6×10^−6^ cm/s), indicating that the 3D BBB chip microenvironment promoted improved barrier function in the cultured brain endothelium (S3A Fig).

Although we did observe some breaks in endothelial monolayer continuity and loss of the permeability in some devices, an intact endothelial barrier was observed in over 85% of the chips. Interestingly, cell layers with large defects that were clearly visible in bright-field microscopy showed diffusion similar to bare collagen, whereas cell layers with minor defects could be easily detected due to localized release of the fluorescent tracer, and permeability values in defective monolayer ranged from 10^−5^ to 10^−4^ cm/s.

A limitation of our 3D BBB chips is that the cylindrical geometry does not allow for TEER measurements because it is not possible to introduce electrodes into the lumen without injuring the surrounding cell layers. We did, however, measure TEER values in the Transwell cultures, which yielded values of 40–50 Ω×cm^2^ (S4 Fig), that while low, are still within the range that has been previously reported for primary human brain endothelium [55]. The TEER values of monocultures of astrocytes and pericytes are in the higher range of what has been reported in literature; however, these cells do not form a tight monolayer with well-formed intercellular junctions and so this resistance is likely due to the high cell densities in these cultures. In our study, when endothelial cells were co-cultured with pericytes or astrocytes, the TEER values were higher than that measured in endothelium alone, but this increased TEER could be accounted for by adding the TEER values of the individual cell types that were present, as no significant synergistic effect was detected when analyzed by one-way ANOVA [55]. While synergistic effects of astrocytes and pericytes on barrier properties of brain endothelium have been reported previously, it is well known that this response varies greatly depending on cell source and culture conditions [55], and our conditions apparently did not support this response.

Taken together, these results show that our 3D BBB chips that were produced with all human primary brain neurovasculature-derived cells display a permeability barrier function that is at least as good as conventional *in vitro* models of the BBB that use non-human cells or immortalized cells. While there have been two prior studies describing dynamic BBB models with all human primary cells, they did not include a realistic 3D ECM or reconstitute direct cell-cell contacts between the different cell types [24, 31], as we did here. Although cylindrical ECM gels have been created in microchannels by viscous fingering and used for cell culture on their inner luminal surface in the past [39], our studies extend this work by demonstrating that a parenchymal cell type (human astrocytes) can be incorporated within the ECM surrounding the vessel-like lumen during its formation. Moreover, the sequential seeding of pericytes and endothelial cells resulted in reconstitution of normal tight associations between endothelial cells and pericytes, which has not been observed previously in BBB cultures. In addition, the circular lumen, the development of extended astrocyte cell processes through the 3D collagen matrix, and the direct interaction of perivascular cells and astrocytes with the endothelial monolayer create a culture microenvironment that more closely resembles the *in vivo* situation, compared to Transwell cultures that are commonly used to model the BBB in vitro.

### 3D blood-brain barrier chips display a unique inflammatory cytokine release profile

Finally, we then used this 3D human BBB-on-a-chip to study the neuroinflammatory response *in vitro*. TNF-α is a pro-inflammatory cytokine implicated in various inflammatory diseases of the central nervous system associated with meningitis, multiple sclerosis, Alzheimer’s disease, AIDS-related dementia, stroke and brain ischemia, among others [56]. While stimulated macrophages and monocytes are primarily responsible for producing systemic circulating TNF-α, several cell types in the brain, including astrocytes, microglia, and even injured neurons, can secrete TNF-α as a paracrine mediator of inflammation. Elevated TNF-α levels in the brain and serum also have been observed in inflammatory diseases of the central nervous system, such as Alzheimer’s disease [57], multiple sclerosis [58] and traumatic brain injury [59].

**Fig 5:**
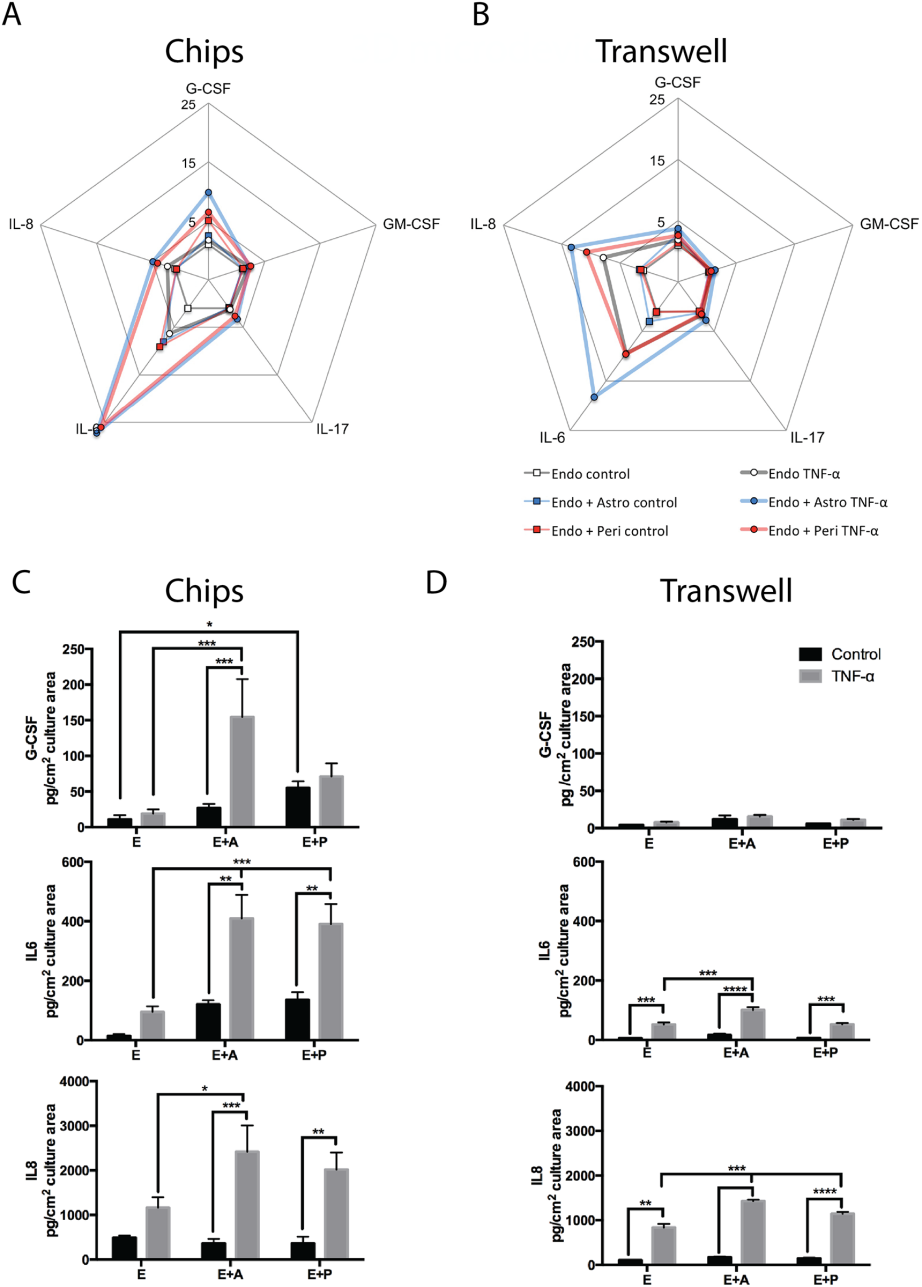
Comparison of cytokine release profiles after inflammatory stimulation with TNF-α in the microfluidic 3D BBB chip versus static Transwell cultures. A-B) Diagrammatic representation of the profile of cytokine release for 5 inflammatory cytokines (G-CSF, GM-CSF, IL-6, IL-8, IL-17) in the 3D BBB chips (A) versus Transwells (B). All data were normalized to the levels of cytokines released by endothelial cells cultured alone; concentric scales indicate fold increase. C-D) Release of G-CSF, IL-6 and IL-8 in the 3D BBB chips (C) and Transwells (D) under basal conditions and when stimulated with TNF-α, normalized for culture area (* p<0.05, ** p<0.01, *** p<0.001, **** p<0.0001 Multiple-comparison ANOVA with Bonferroni's comparisons test; n = 4–7 for 3D BBB chips and n = 3 for Transwells).

To explore whether we can use the synthetic nature of the 3D BBB chip to analyze the contributions of individual brain vasculature-associated cells to neuroinflammation, we cultured the engineered microvessels in the presence or absence of TNF-α (50 ng/ml) that was flowed through the lumen for 6 hours. We then analyzed cytokine release profiles produced in the 3D BBB chips containing endothelium with or without either pericytes or astrocytes, and we compared these results to those obtained with similar mono-cultures, as well as co-cultures maintained in commercial Transwell culture plates. Of the seventeen cytokines tested (see Materials and Methods), five exhibited a detectable and consistent release pattern in the 3D BBB chips: granulocyte colony stimulating factor (G-CSF), granulocyte macrophage colony stimulating factor (GM-CSF), interleukin-6 (IL-6), interleukin-8 (IL-8/CXCL8), interleukin-17 (IL-17). Comparison of the release profiles of these five cytokines normalized relative to their release from the unstimulated endothelium revealed that secretion of G-CSF and IL-6 were significantly different in 3D BBB chips compared to conventional Transwell co-cultures (Fig 5A and 5B, S5 Fig). Quantitative comparisons also showed that secretion levels of G-CSF, IL-6 and IL-8 were significantly higher in the microfluidic BBB chip compared to static Transwell cultures, and this difference was most pronounced with G-CSF and IL-6 (Fig 5C and 5D). Use of the BBB chip also revealed that astrocytes and pericytes can independently enhance the secretion of G-CSF and IL-6 when co-cultured with endothelium even under basal unstimulated conditions, whereas this was not detected in the Transwell system (Fig 5C and 5D). The fold increase in IL-6 and IL-8 secretion induced by TNF-α was also higher in Transwell cultures than in BBB chips (S5 Fig), which may be partially explained by the higher basal levels of secretion of these cytokines in the chips. In contrast, the induction of G-CSF was more pronounced in 3D BBB chips than in Transwells, and in fact, the levels of this cytokine were almost undetectable in these planar cultures (S5 Fig).

Our ability to detect changes in G-CSF levels in the 3D BBB chip provides a significant advantage over Transwell BBB models for studies on neuroinflammation as G-CSF is an important neuroprotective cytokine [60] secreted in response to brain injury by endothelial cells, astrocytes and neurons [61–63]. G-CSF promotes neuronal survival and proliferation [63], in addition to stimulating recruitment of bone marrow-derived endothelial progenitor cells that stimulate vascular repair [64]. Animal experiments also have shown that exogenously administered G-CSF can inhibit neuronal cell death after ischemic brain injury [65, 66]. Thus, it is interesting that we observed similar strong TNF-α-mediated induction of G-CSF secretion in our 3D co-culture model of brain endothelial cells and astrocytes under microfluidic conditions whereas this could not be detected when the same cells were co-cultured under static conditions in Transwells. Interestingly, however, because we could independently study the contributions of pericytes and astrocytes to this response, we discovered that the presence of pericytes was alone sufficient to increase baseline levels of G-CSF secretion in the 3D BBB chip model, and these cultures were not sensitive to induction by TNF-α. In contrast, 3D BBB chips containing astrocytes and endothelial cells exhibited up to a 10-fold increase in G-CSF secretion in response to TNF-α stimulation.

IL-6, which is strongly expressed by neuronal, glial and vascular tissue during neuroinflammation *in vivo*, modulates both the acute and late-stage immune responses [67, 68]. Acutely it prevents neuronal injury by protecting against apoptosis due to oxidative stress and controls the innate immune response that is mediated by neutrophils and monocytes [69], whereas in later stages of neuroinflammation, IL-6 stimulates angiogenesis and re-vascularization [70, 71]. Levels of secreted IL-6 also correlate with brain infarct size in ischemic stroke [72] and high IL-6 levels are associated with a negative functional outcome after traumatic brain injury [73]. Importantly, we observed a similar response to the inflammatory stimulus TNF-α in our 3D BBB chip co-cultures, with strong IL-6 induction in co-cultures of both astrocytes-endothelial cells and pericytes-endothelial cells, whereas these responses were barely detectable in Transwell cultures (Fig 5A–5D).

IL-8 is an activating and pro-inflammatory cytokine produced by astrocytes, pericytes and endothelial cells that is primarily involved in recruiting neutrophils to sites of injury [74–77]. Levels of IL-8 are markedly increased in the context of neural injury [78] and inhibition of IL-8 signaling is associated with improved outcome in the context of neuroinflammation [79, 80]. While both our 3D BBB chip and Transwell cultures demonstrated enhanced IL-8 production in response to TNF-α stimulation when astrocytes or pericytes were present in combination with endothelial cells, our 3D BBB chip co-cultures again showed a greatly enhanced level of response in terms of the absolute amount of cytokine that was produced (Fig 5C and 5D).

Another major difference between our 3D BBB microfluidic chip and Transwell cultures, as well as past microfluidic BBB models, is that these other models contain semi-permeable membranes that separate the interacting cell types [22, 26, 27, 81–83]. These membranes are typically rigid thick (10–50 μm) substrates with pores (0.4–3 μm diameter) that constitute an artificial barrier between the neurovascular cells. In contrast, in the 3D BBB chip, we utilized a compliant ECM gel constrained within a confined cylindrical geometry and positioned the endothelial cells, pericytes and astrocytes in ways that allowed them to reconstitute their normal 3D spatial relationships and reestablish more natural cell-cell interactions, resulting in deposition of an intervening type IV collagen-containing basement membrane. At the same time, it is important to note that the 3D BBB chip does not fully recapitulate the *in vivo* situation in that the endothelial cells were not subjected to continuous fluid flow and physiologically relevant levels of shear stress during their entire 5 day culture period; however, we did expose the cells to continuous flow when we analyzed their permeability barrier and neuroinflammatory responses (cytokine secretion profiles). It is important to note that most previously reported microfluidic models of the BBB similarly fail to include realistic levels of shear stress during sustained culture, probably for similar reasons (the cost of using large amounts of culture medium). For example, a recent review of microfluidic BBB models found that only three out of twelve studies included physiological levels of shear stress for at least part of their experiments [84]. Other limitations of our model are that the lumen of the 3D BBB chip is almost an order of magnitude larger than that of a typical brain microvessel, and the pericytes and astrocytes processes form contacts with a smaller fraction of the endothelium on-chip than in living brain capillaries. However, as all of these features can be controlled and varied in an independent manner using this microengineered approach, it should be possible to determine their relative importance for BBB structure and function in future studies. Our data show that this 3D BBB chip reconstitutes more normal spatial relationships and provides a more balanced and physiologically relevant picture of human neurovascular inflammation *in vitro* than static Transwell cultures, as demonstrated by enhanced secretion of both pro-inflammatory (IL-6) and neuroprotective (G-CSF) cytokines. As the system utilizes all primary human brain-derived cells in addition to mimicking the 3D architecture of the brain microvessel, it also offers an advantage over previously described 2D microfluidic systems that both lacked this structure and utilized non-human or immortalized cells.

## Conclusion and Outlook

This method for establishing co-cultures of multiple types of primary human brain-derived vascular cells (endothelial cells, pericytes and astrocytes) in microfluidic chips that reconstitute their normal 3D spatial relationships has permitted us to dissect the contributions of these cells to the neuroinflammatory response *in vitro*. We first showed that a viscous fingering method can be used to create cylindrical compliant collagen gels within a microfluidic channel, and that hydrostatic pressure-driven flow can be used to control the dimensions of the lumen without having to adjust the channel dimensions or the viscosity of the collagen solution. Using this configuration, multiple modes of co-culture were then established by either embedding astrocytes inside the gel or by performing sequential seeding of pericytes and endothelial cells inside the lumen. The reconstituted all human 3D BBB-on-a-chip formed a permeability barrier similar to that previously reported for cultured non-human or immortalized cells, and the integrity of the endothelium was found to strongly depend on the presence of astrocytes and pericytes in the cultures. Finally, we demonstrated that the BBB chips that contained these neurovascular cells and reconstituted their normal 3D cell-cell relationships exhibited responses to an inflammatory stimulus (TNF-α) that more closely mimicked those observed in the living brain than the same cells when co-cultured in a planar static Transwell culture. Because this is a synthetic system, additional cell types may be integrated in the 3D BBB chip to create more complex co-cultures in the future, including human immune cells, such as neutrophils, microglia and monocytes, as well as human cortical neurons, in addition to the three neurovascular cell types used in the present study. Taken together, these findings suggest that the 3D microfluidic BBB chip that we described here may be suitable to study the vascular component of neuroinflammation and other neurological disorders, as well as to help identify new drugs that target these responses.

## Supporting Information

S1 Fig Marker expression in human primary cells used to populate the 3D BBB chip. Human cerebral cortex microvascular endothelial cells express VE-cadherin (A) and the tight junction protein ZO-1 (B) at intercellular adherens junctions. Human astrocytes display differential expression of glial fibril acidic protein (GFAP) (C) and human brain-derived pericytes express alpha smooth muscle actin (α-SMA) (D), but lack the endothelial markers, VE-Cadherin (E) and PECAM (F), and they clearly do not form a continuous monolayer when the same cells shown in F were stained with phalloidin (G). The staining for each specific marker is shown in magenta; green indicates F-actin stained with phalloidin; blue indicates Hoechst-stained nuclei (bar, 50 μm).
(TIF)

S2 Fig Co-culture of human brain microvascular endothelial cells and pericytes in the 3D BBB chip. Perspective view of brain microvascular endothelium with prior plating of brain pericytes on the surface of the gel in the central lumen. VE-cadherin in magenta and F-actin in green (bar, 200 μm).
(TIF)

S3 Fig Apparent permeability values of human brain microvascular endothelial cells, astrocytes and pericytes in static Transwell cultures. P_app_ values were evaluated using 5 min assay with 3 kDa Dextran after 120 hrs of culture, n = 3.
(TIFF)

S4 Fig TEER values of human brain microvascular endothelial cells, astrocytes and pericytes in static Transwell cultures. TEER values were recorded after 120 hrs of culture, n = 3.
(TIFF)

S5 Fig Comparison of cytokine release profiles after inflammatory stimulation with TNF-α in the microfluidic 3D BBB chip versus static Transwell cultures. All data represent the levels of cytokines released after TNF-α stimulation normalized to the basal condition for each specific culture. E, endothelial cells alone; E+A, co-culture of endothelial cells and astrocytes; E+P, co-culture of endothelial cells and pericytes (* p<0.05 Pairwise Microdevice-Transwell comparison t-tests with Sidak-Bonferroni method for multiple comparisons; n = 4–7 for 3D BBB chips and n = 3 for Transwells).
(TIFF)

S1 Movie 3D reconstruction of confocal micrographs showing the 3D BBB chip with brain microvascular endothelial cells lining the lumen (green,VE-Cadherin; blue, Hoechst-stained nuclei). The width of each frame in the movie is 550 μm.
(MP4)

S2 Movie 3D reconstruction of confocal micrographs showing the 3D BBB chip with human astrocytes embedded in the collagen gel surrounding a lumen lined with brain microvascular endothelial cells (red, F-actin; blue, Hoechst-stained nuclei). The width of each frame in the movie is 550 μm.
(MP4)
