# Supplementary figures and images for: Distinct Contributions of Astrocytes and Pericytes to Neuroinflammation Identified in a 3D Human Blood-Brain Barrier on a Chip
Source: PLoS One. 2016 Mar 1;11(3):e0150360. doi: 10.1371/journal.pone.0150360 (PMC4773137; doi:10.1371/journal.pone.0150360)

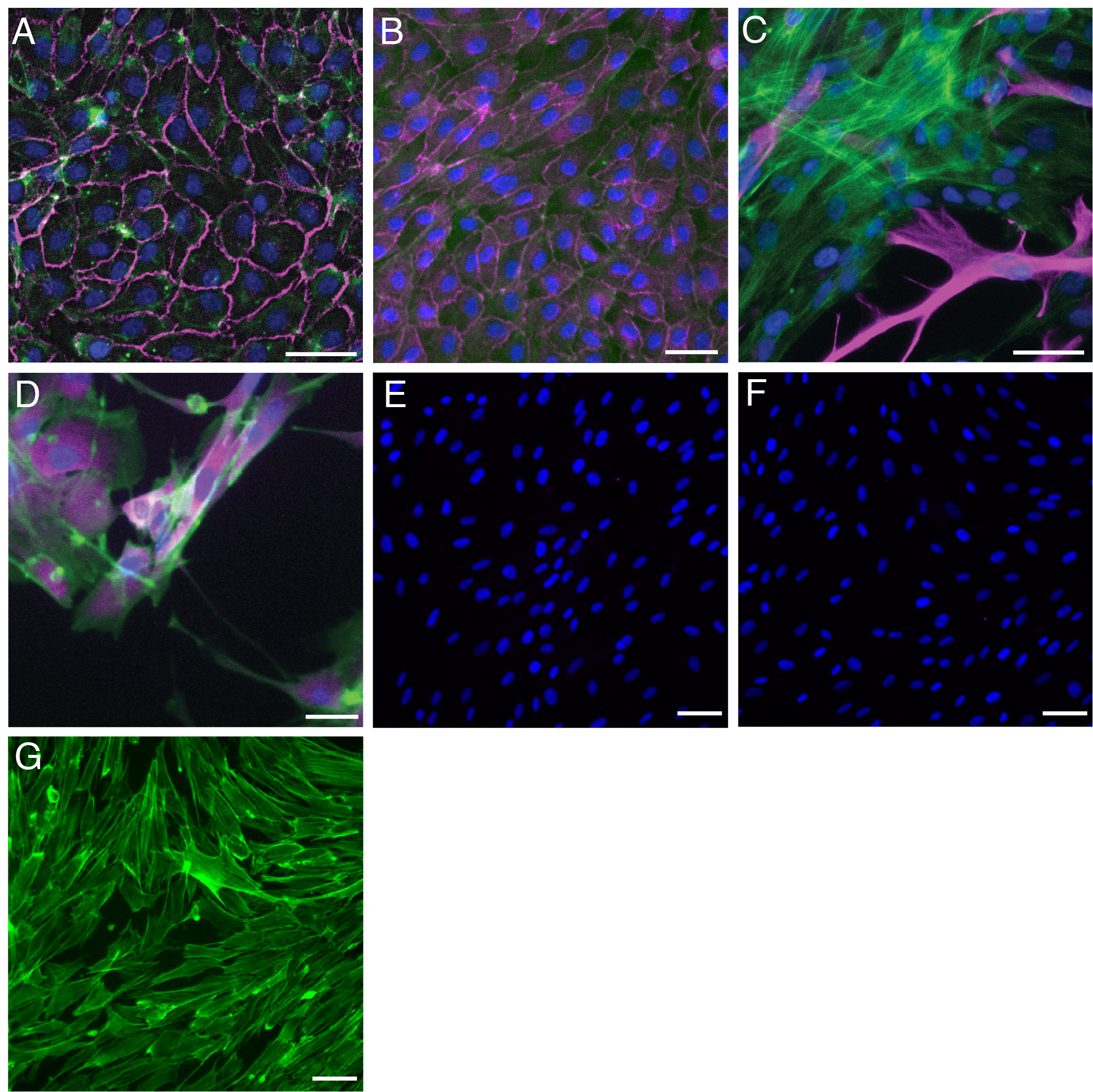

Supplement: S1 Fig — Human cerebral cortex microvascular endothelial cells express VE-cadherin (A) and the tight junction protein ZO-1 (B) at intercellular adherens junctions. Human astrocytes display differential expression of glial fibril acidic protein (GFAP) (C) and human brain-derived pericytes express alpha smooth muscle actin (α-SMA) (D), but lack the endothelial markers, VE-Cadherin (E) and PECAM (F), and they clearly do not form a continuous monolayer when the same cells shown in F were stained with phalloidin (G). The staining for each specific marker is shown in magenta; green indicates F-actin stained with phalloidin; blue indicates Hoechst-stained nuclei (bar, 50 μm). (TIF) [file pone.0150360.s001.tif]

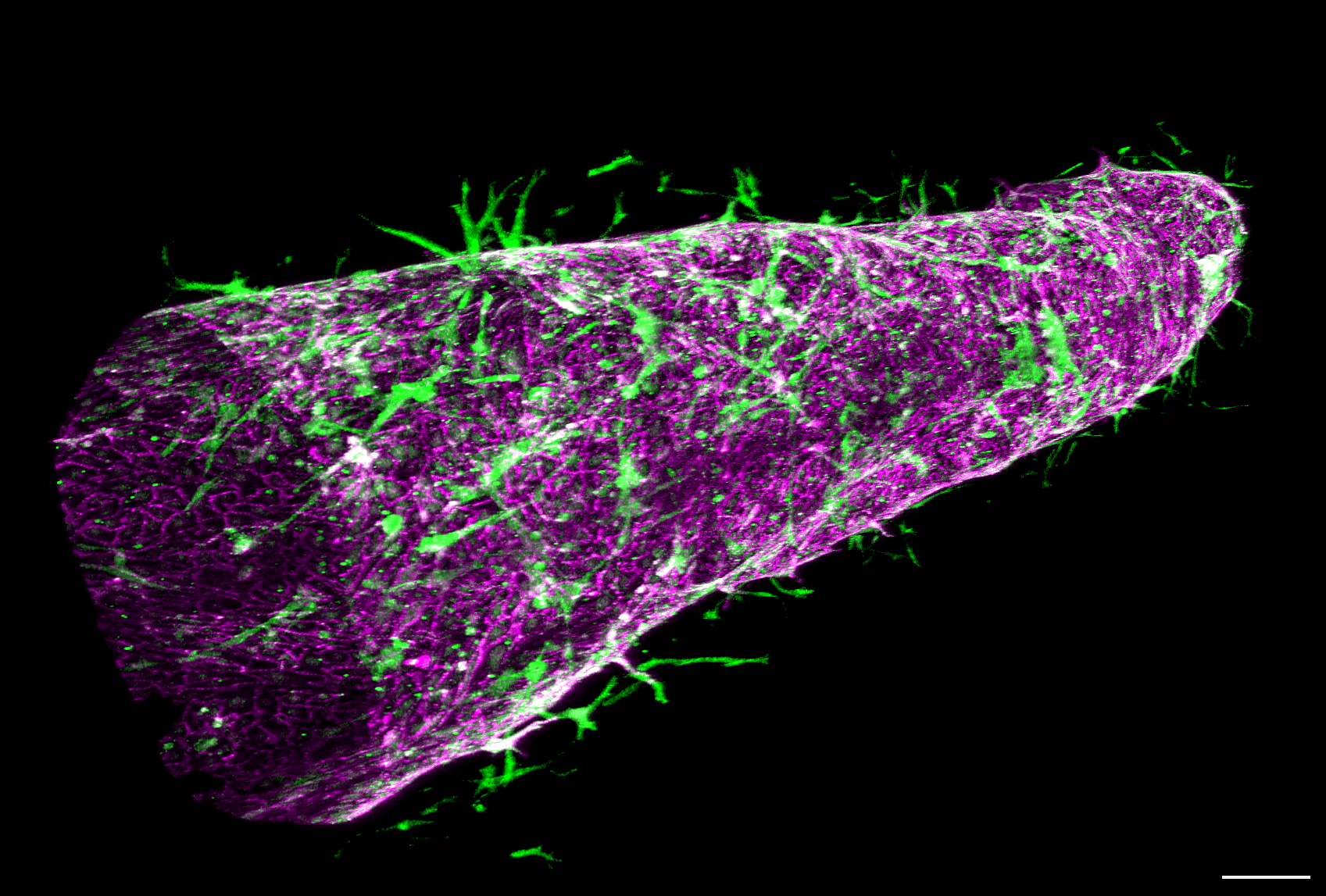

Supplement: S2 Fig — Perspective view of brain microvascular endothelium with prior plating of brain pericytes on the surface of the gel in the central lumen. VE-cadherin in magenta and F-actin in green (bar, 200 μm). (TIF) [file pone.0150360.s002.tif]

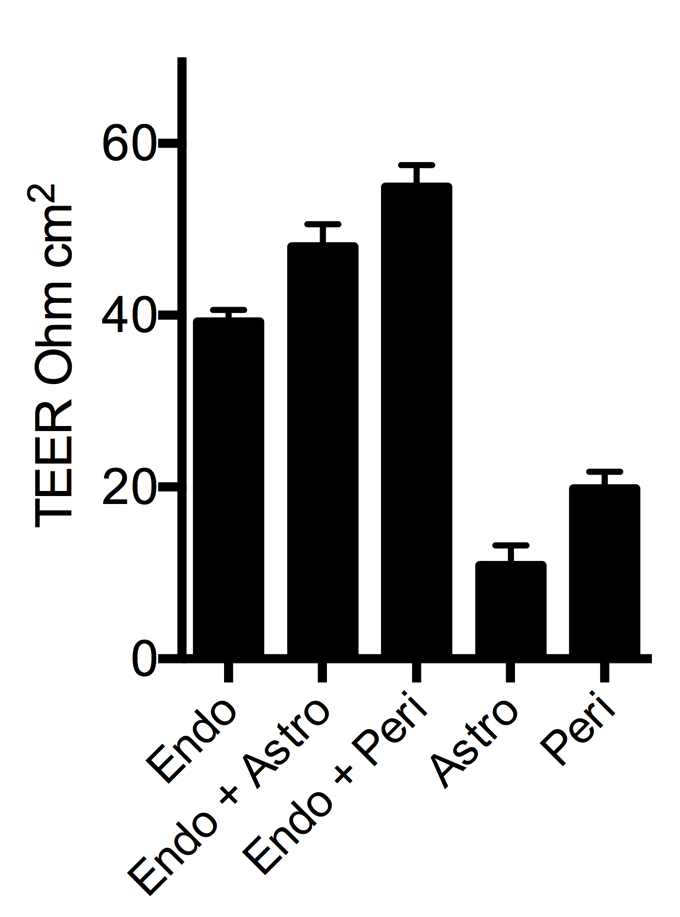

Supplement: S4 Fig — TEER values were recorded after 120 hrs of culture, n = 3. (TIFF) [file pone.0150360.s004.tiff]

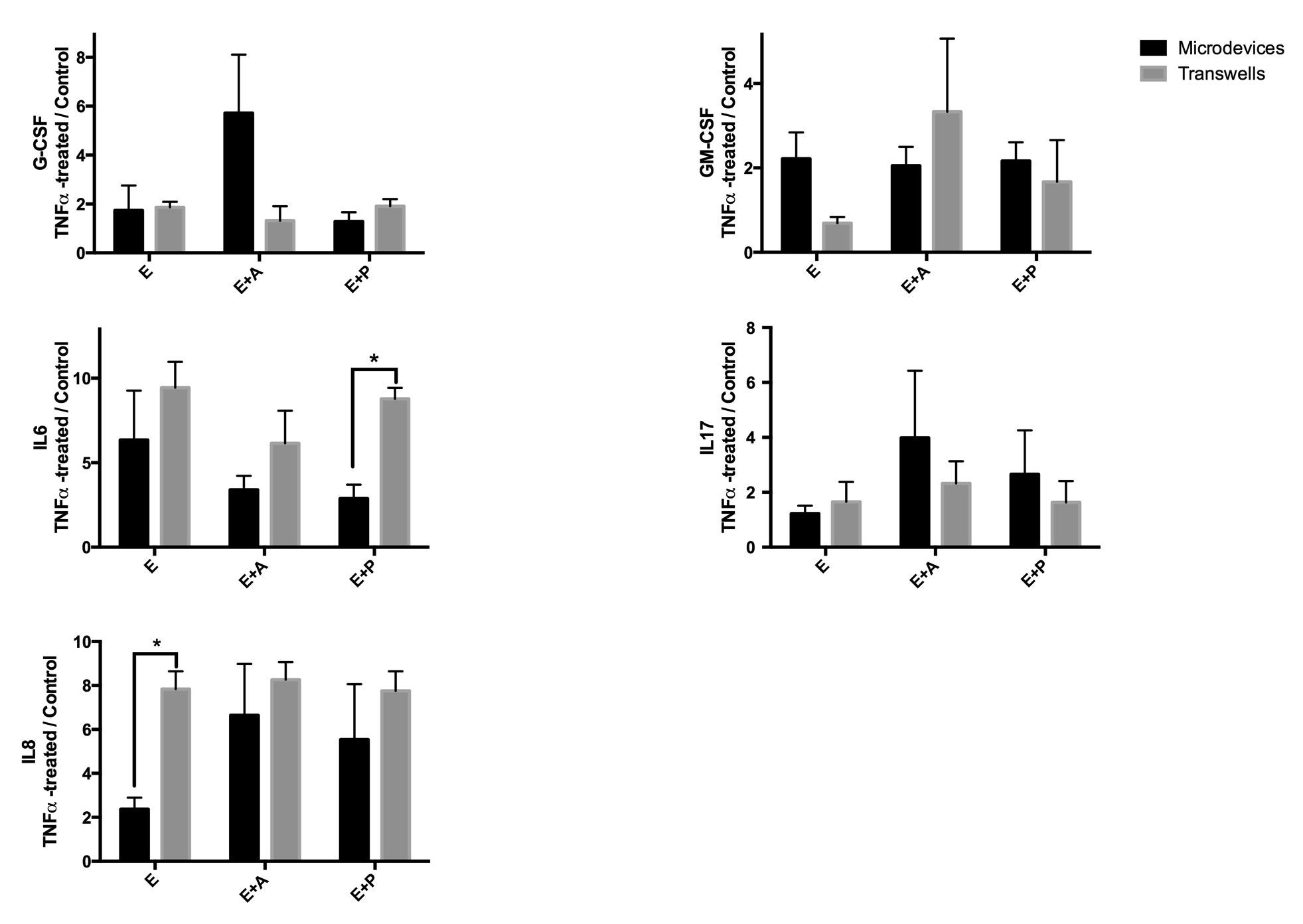

Supplement: S5 Fig — All data represent the levels of cytokines released after TNF-α stimulation normalized to the basal condition for each specific culture. E, endothelial cells alone; E+A, co-culture of endothelial cells and astrocytes; E+P, co-culture of endothelial cells and pericytes (* p<0.05 Pairwise Microdevice-Transwell comparison t-tests with Sidak-Bonferroni method for multiple comparisons; n = 4–7 for 3D BBB chips and n = 3 for Transwells). (TIFF) [file pone.0150360.s005.tiff]
